# Prognostic Impact of Severe Atrial Functional Tricuspid Regurgitation in Atrial Fibrillation Patients

**DOI:** 10.3390/jcm11237145

**Published:** 2022-12-01

**Authors:** Ancuța Elena Vîjan, Ioana Cristina Daha, Caterina Delcea, Elisabeta Bădilă, Gheorghe-Andrei Dan

**Affiliations:** 1 Internal Medicine and Cardiology Department, Carol Davila University of Medicine and Pharmacy, 020021 Bucharest, Romania; 2 Cardiology Department, Colentina Clinical Hospital, 020125 Bucharest, Romania

**Keywords:** tricuspid regurgitation, atrial functional tricuspid regurgitation, atrial fibrillation, mortality

## Abstract

Background and Aim: Atrial fibrillation (AF) is an epidemic disease with a significant global health impact. Atrial functional tricuspid regurgitation (AF-TR) is a more recently acknowledged complication of AF. The main purpose of this study was to determine the prognostic value of severe AF-TR in patients with AF, and its determinants. Methods: In this retrospective, observational study, we included AF patients admitted consecutively to a tertiary clinical hospital between January 2018 and February 2020, irrespective of cause of hospitalization. Patients with organic TR, significant pulmonary hypertension, left ventricular ejection fraction < 50%, those with implanted cardiac devices and those with in-hospital mortality were excluded. Severe TR was defined according to current guidelines. Median follow-up time was 34 (28–39) months. Primary endpoint was all-cause mortality. Results: We included 246 AF patients, with a mean age of 71.5 ± 9.4 years. 86.2% had AF-TR, while 8.1% had severe AF-TR. Mortality rate was 8.5%. Right atrial diameter (*p* = 0.005), systolic pulmonary artery pressure (sPAP) (*p* = 0.015) and NT-proBNP (*p* = 0.026) were independent predictors for the presence of severe valvular dysfunction. In multivariable survival analysis, severe AF-TR, was an independent predictor of all-cause mortality (HR 5.4, 95% CI 1.1–26.2, *p* = 0.035). Conclusion: Severe AF-TR was an independent predictor of mortality in AF patients, while mild/moderate AF-TR apparently had no impact on prognosis.

## 1. Introduction

Atrial fibrillation (AF) is an epidemic disease burdening global public health [1]. The most common hospitalized arrhythmia in clinical practice, it is associated with increased cardiovascular morbi-mortality [2]. Even after adjusting for other cardiovascular conditions, AF portends an independent risk for mortality [3]. With a prevalence on the rise due to better survival and ageing of the population, it is estimated that one in 4–6 adults will develop AF during their life [4,5]. Although mortality trends have been decreasing lately, the health and financial burden of AF, its complications and related hospitalizations remains high [1,6]. In anticoagulated AF patients, the most common causes of death were heart failure (HF), infections and cancer, with only 7% of deaths secondary to stroke [7].

A long-term complication of AF is represented by functional atrioventricular regurgitation. In AF patients the remodeling process involving the atrium, ventricle and valvular apparatus can lead to significant valvular insufficiency [8]. Most studies focusing on functional valvular regurgitation have focused on the spotlight on the mitral valve, while data regarding the functional tricuspid regurgitation in AF are scarce.

Isolated functional TR without significant left heart or pulmonary pathologies was described mainly in older populations with an estimated prevalence of 8–9% [9,10]. AF is considered to be one of the main causes of atrial functional TR (AF-TR) [11]. The direct correlation of tricuspid annular dilation and AF-TR was described in 2011, when Spinner et al. proved that 40% right atrium (RA) dilation was sufficient to determine significant TR in vitro [12]. The theory was confirmed soon after, when Najib et al. identified the larger dimensions of both RA and right ventricle (RV) as independent predictors of severe AF-TR, alongside older age and chronic AF, drivers of right heart remodeling [13]. However, interest in the topic has remained low until very recently.

Pathophysiologically, functional TR in AF patients occurs mainly as a result of tricuspid annulus remodeling, RA dilatation and loss of function, despite preserved RV function [14]. The RV develops isolated inflow dilation, accentuating the enlargement of the tricuspid annulus, and thus participating in the development of AF-TR [15]. Despite having similar vena contracta, AF-TR had less leaflet tethering and larger tricuspid annulus with more posterior orientation [16]. 3D-studies of the tricuspid valve in AF-TR patients showed differences in tricuspid annulus geometry and size depending on the severity of the tricuspid regurgitation. Functional tricuspid annulus parameters were similar regardless of the grading of tricuspid regurgitation [17].

Significant AF-TR is associated with higher grades of right atrial dysfunction and lower values of right atrial strain, reinforcing the concept that AF-TR is mainly a disease of RA and tricuspid annulus [18].

Previous research regarding the prognosis of atrial functional regurgitation were centered on functional mitral regurgitation. Abe et al. showed that the combination of mitral and tricuspid regurgitation secondary to AF was associated with increased mortality [19].

The impact of severe AF-TR on mortality in AF patients is poorly defined. Although mild TR does not alter the outcome, few studies found that moderate to severe TR of different etiologies was associated with increased mortality [10,20]. Therefore, the main purpose of this study is to determine the survival prognostic value of severe AF-TR in patients with AF. Secondary objectives evaluate the main determinants of severe AF-TR.

## 2. Material and Methods

### 2.1. Population

This is a retrospective, observational study. We evaluated AF patients admitted to a tertiary clinical hospital in the cardiology ward between January 2018 and February 2020. The study protocol was approved by the Hospital’s Ethical Board in accordance with the Declaration of Helsinki.

We included all AF patients admitted consecutively to the Cardiology Department, according to the criteria previously published [6]. Patients with organic TR, significant pulmonary hypertension, left ventricular ejection fraction < 50%, those with implanted cardiac devices [17,21] and those with in-hospital mortality were excluded. Rehospitalizations of the same patient were also excluded.

Median follow-up time was 34 (28–39) months.

Primary endpoint was all-cause mortality, which was evaluated in October 2021.

### 2.2. Definitions

Atrial functional TR was defined as the following: TR without significant pulmonary hypertension (systolic pulmonary artery pressure (sPAP) < 50 mmHg) and no evident TR cause (left ventricle ejection fraction <50%, organic tricuspid disease, other significant valvular disease, pacemaker/defibrillator wire inserted over the tricuspid valve, or pericardial disease) and no previous valve surgery [21]. AF patients were classified in two groups: severe TR and non-severe TR. Non-severe TR included no TR, mild and moderate TR. The severity of TR was defined according to the actual 2017 ESC guidelines: qualitative (tricuspid valve morphology, color flow TR jet, continuous wave Doppler signal of TR jet) or semi-quantitative criteria (vena contracta width > 7 mm, proximal isovelocity surface area radius >9 mm, systolic flow reversal of the hepatic vein flow, tricuspid inflow with dominant E-wave ≥ 1 m/s) [22].

sPAP was determined by calculating the sum of the estimated right atrial pressure and tricuspid regurgitant jet gradient. Right atrial pressure was considered 5 mmHg if the inferior vena cava (IVC) was less than 21 mm in diameter, 10 mmHg if IVC was dilated and collapsed with respiration and 15 mmHg if the IVC was dilated and did not collapse with respiration [20].

AF was classified in three groups: paroxysmal, persistent, and permanent.

CHA_2_DS_2_-VASc score was calculated for all patients in accordance with the ESC guidelines [2].

eGFR (estimated glomerular filtration rate) was obtained using the CKD-EPI formula.

### 2.3. Laboratory Measurements

General Electrics Vivid S6 and Philips Epiq 7 (KPI Healthcare) were used to perform transthoracic echocardiography by experienced staff physicians. Qualitative (color flow regurgitant jet continuous wave Doppler) and quantitative (regurgitant volume) measurements were performed to evaluate the TR.

### 2.4. Statistical Analysis

The statistical analysis was performed using SPSS 19, Medcalc, EpiInfo 2007.

Descriptive statistics were reported as absolute numbers and percentages for categorical variables. Non-parametric data were expressed as median with interquartile range and normally distributed data as mean with standard deviation. Independent continuous data were compared using Anova and Mann–Whitney–Wilcoxon tests. Mortality rates were assessed by Kaplan Meyer curves.

ROC curve analysis was performed to test the correlations between numerical and categorical variables.

Multivariable logistical regression, Enter method, was used to determine independent predictors for AF-TR. All variables with statistical significance identified in univariate analysis were included in the regression.

Cox proportional hazards multivariable analysis consisted of two regressions: the first analysis included all parameters associated with mortality in univariable analysis to determine independent predictors; the second analysis included the identified independent predictors and severe AF-TR.

*p* value < 0.05 was considered to be the cutoff for statistical significance.

## 3. Results

**Figure 1:**
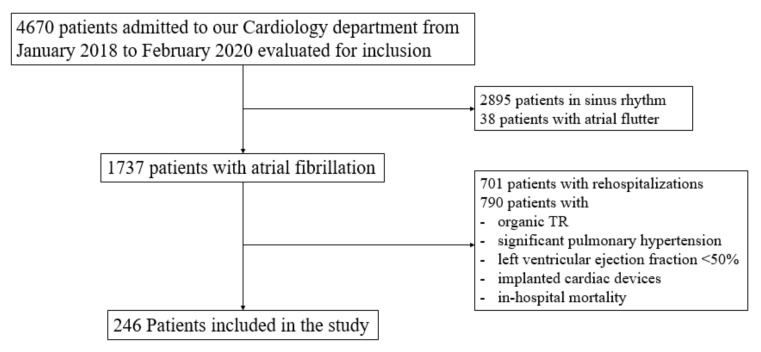
PRISMA consort flow diagram of the study.

Our study included 246 patients with AF (Figure 1).

Mean age was 71.5 ± 9.4 years. In total, 63.0% of them were women. Among the group, AF-TR had a prevalence of 86.2%, while 8.1% had severe AF-TR. All-cause mortality for the entire study group was 8.5 % and 25.0% for patients with severe AF-TR during a median follow-up time of 34 (28–39) months.

The prevalence of HF (74.4%), arterial hypertension (87.4%) and dyslipidemia (80.1%) was increased in the study group. Other associated comorbidities were ischemic heart disease (IHD), stroke or transitory ischemic attack, diabetes mellitus, chronic kidney disease, dementia and obesity (Table 1). Of the three types of AF, persistent and permanent AF had similar prevalence, being more frequent compared to paroxysmal AF.

Both left and right atrial dimensions were linked to the type of AF, from the smallest values for paroxysmal and the largest values for permanent AF (*p* for trend < 0.001, Table 2). The same increasing tendency was noted for right ventricular dimensions (*p* for trend < 0.001), as well as sPAP (*p* for trend < 0.001) (Table 2).

### 3.1. Characteristics of Patients with Severe AF-TR

Patients with severe AF-TR were older (*p* for trend: 0.005) with more comorbidities and higher NYHA class (25.0%). Non-paroxysmal AF (65%) was prevalent in this group, while paroxysmal AF (41.6%) was more frequent in patients with non-severe AF-TR (Table 1). Regarding the echocardiographic parameters, RA (*p* < 0.001) and RV diameters (*p* < 0.001) were significantly higher in the severe AF-TR group with increased mean SPAP values (*p* < 0.001) (Table 1). Severe AF-TR was directly correlated with RA (AUC 0.81, *p* < 0.001) and RV diameters (AUC 0.80, *p* < 0.001) and sPAP (AUC 0.82, *p* < 0.001) (Table 3).

Of the clinical and laboratory parameters associated with severe AF-TR in univariate analysis (Table 3), the RAD (HR 1.11, *p* = 0.005), sPAP (HR 1.20, *p* = 0.015) and NT-proBNP (HR 2.37, *p* = 0.026) were independent predictors for the presence of severe valvular dysfunction in multivariable analysis (Table 4).

### 3.2. Severe AF-TR and Mortality

In univariate analysis, severe AF-TR was a predictor of all-cause mortality in AF patients (OR 4.37, *p* = 0.005). Other variables associated with all-cause mortality were ADHF (OR 4.32, *p* < 0.001), NYHA class III orIV (OR 5.74, *p* < 0.001), TIA/stroke (OR 2.81, *p* = 0.04), dementia (OR 6.08, *p* = 0.006) and infection (OR 5.27, *p* < 0.001) (Table 5).

In ROC analysis, age (AUC 0.77, *p* < 0.001), NT-proBNP levels (AUC 0.80, *p* < 0.001), RVD (AUC 0.64, *p* = 0.05) and creatinine (AUC 0.66, *p* = 0.02) were directly predictive of all-cause mortality, while ejection fraction was inversely correlated with this endpoint (AUC 0.69, *p* = 0.009) (Table 5).

**Figure 2:**
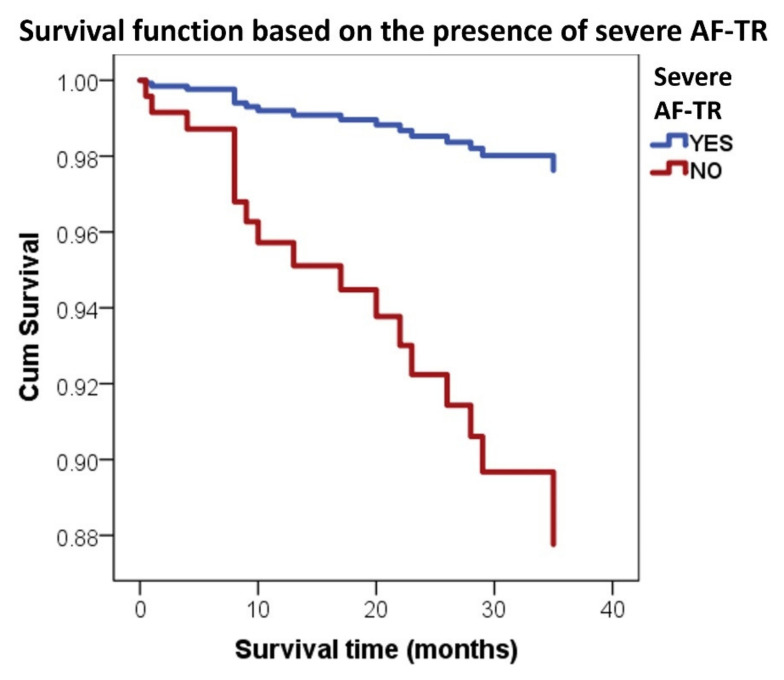
Survival analysis of patients with and without severe AF-TR.

In multivariable Cox proportional hazards survival analysis, severe AF-TR (HR 5.44, *p* = 0.035) (Figure 2), dyspnea at mild effort or at rest (HR 3.9, *p* = 0.016), infections (HR 3.79, *p* = 0.017), age (HR 1.07, *p* = 0.029) and LVEF (HR 0.82 for inverse correlation, *p* = 0.008) were independent predictors of all-cause mortality in AF patients (Table 6).

## 4. Discussion

Our study is among the scarce proof of the independent prognostic impact of AF-TR in AF patients

Until recently, TR was an underestimated and neglected entity, considered almost exclusively a consequence of left heart disorders or pulmonary disease, without intrinsic prognostic value. Currently, there is a positive trend toward recognition of the AF-TR in AF patients and its prognostic value [23,24,25,26].

In our analysis, severe AF-TR in AF patients had a prevalence of 8.1%. Two previous studies showed a prevalence of so called significant AF-TR of 21.7 % and, respectively, 15% [19,23], which can be explained by the inclusion of patients with both moderate as well as severe AF-TR in these studies.

Previous studies showed that AF-TR is a common finding in patients with non-paroxysmal AF mainly due to bi-atrial enlargement and tricuspid annular dilation [27]. In our analysis, patients with severe AF-TR were older, similar to results from previous studies [24,28].

### 4.1. Atrial Functional Tricuspid Regurgitation

In 2020, from their retrospective analysis of a large cohort of 1552 patients, Mutlak et al. concluded that the progression of AF-TR is driven by age, AF and high left-sided filling pressures characterized by increased PAP and LA enlargement [29]. Our results confirm that severe AF-TR patients are older, more likely to have permanent AF and a higher burden of comorbidities expressed by the CHA2DS2-VASc score, as well as larger LA dimensions and higher sPAP. Moreover, sPAP and NT-proBNP values were independent predictors of severe AF-TR in multivariable analysis, alongside the RA diameter. The correlation of RA dimensions and AF-TR was also reinforced by Utsunomiya et al. [9] and Dietz et al. [24].

Better characterization of the TV anatomy and pathophysiology of AF-TR was achieved lately with the study of Guta et al. using three-dimensional echocardiography to assess the RA remodeling process in long-standing persistent AF patients [17]. The authors concluded that the role of RA dilation surpasses that of RV enlargement to induce AF-TR [17]. Our data reinforce the association of severe AF-TR with RA diameter. In univariable analysis, severe AF-TR patients had larger dimensions of both RA and RV; however, only RA diameter was an independent predictor for the severe valvular insufficiency in multivariable analysis.

### 4.2. Severe AF-TR and Mortality of AF Patients

AF patients’ survival has shown improvement lately, given mainly to the advances in the prevention of thrombo-embolic events; however, these individuals still have increased mortality compared to those in sinus rhythm [30]. Recent data linked significant AF-TR with negative outcomes in AF patients. Dietz et al. correlated moderate and severe AF-TR with the composite end-point of all-cause mortality and hospitalization for heart failure and stroke during a median follow-up of 62 months [24]. Prapan et al. also found moderate to severe TR to predict the occurrence of heart failure or death from any cause during a two-year follow-up [23]. In another selected AF cohort with at least moderate TR, during a median period of 52 months, the severity of AF-TR was indicative of all-cause mortality [25]. A post-hoc analysis of MISOAC-AF trial analyzing valvular heart disease in AF patients also confirmed the association of TR with mortality [26].

The particularity of our research compared to the aforementioned studies is the independent correlation of severe AF-TR with all-cause mid-term mortality, after adjustment for identified survival predictors in our cohort. Both Dietz et al. [24] and Prapan et al. [23] evaluated composite end-points as adverse events, not mortality alone. Fortuni et al. [25] referred to only a subset of severe TR, respectively, torrential AF-TR, as a driver of mortality, while Samaras et al. [26] evaluated severe TR of all etiologies, not specifically AF-TR.

Our study correlated the prevalence of severe AF-TR with persistent and permanent AF and subsequently increasing RA dimensions due to atrial remodeling. The independent mortality impact of AF-TR in these patients could therefore be indirect proof for early rhythm control and the superiority of the rhythm control strategy.

In 2020, evidence emerged showing that in patients with persistent AF, successful rhythm strategy can improve the RA geometry and therefore the severity of AF-TR. A case series of two AF patients that regained sinus rhythm reported by Muraru et al. [27] and a retrospective cohort study of persistent AF patients undergoing catheter ablation published by Itakura et al. proved that the rhythm strategy can induce reverse remodeling of the RA with subsequent correction of AF-TR severity [31].

Similar findings were also reported regarding functional mitral regurgitation in AF patients [32]. These results confirm that the benefit of rhythm strategy in AF patients may surpass that of symptomatic relief and extend to the improvement in morbi-mortality. In-vitro data support the premise that AF-TR is highly dependent on the tricuspid annulus geometry, developing after only 40% dilation, compared to fMR which requires a 75% increase in mitral annulus diameter [12,33].

## 5. Limitations

This study has several limitations. First, it is a retrospective observational study, conducted in a single center. Moreover, the study was performed on a small sample, yet representative for the diversity of AF patients.

Another important limitation was related to the echocardiographic evaluation of the tricuspid regurgitation and RV dimensions and function, including the lack of 3D echocardiography. As mentioned before, the TR grading was based on qualitative and semi-quantitative parameters.

## 6. Conclusions

Atrial functional TR is a common finding in AF patients. Severe AF-TR was an independent predictor of mortality in AF patients, while mild/moderate AF-TR had no impact on prognosis. Severe AF-TR was determined by increased RA diameter and sPAP and correlated with higher NT-proBNP values.

Severe AF-TR was highly prevalent in non-paroxysmal AF patients, raising the hypothesis that an aggressive rhythm strategy could prevent permanent atrial remodeling and therefore the progression of TR severity with a potential prognostic benefit.

## Figures and Tables

**Table 1:** Baseline characteristics of the study group.

	Total N = 246	Non-Severe TR * N = 226	Severe TR ** N = 20	*p* Value for Comparison of * and **
Demographics				
Age, years	71.5 ± 9.4	71 ± 9.4	77 ± 7.7	0.005
Women	155 (63.0%)	140 (62.0%)	15 (75.0%)	0.25
Heart failure characteristics				
HF	183 (74.4%)	165 (73.0%)	18 (90%)	0.09
ADHF	65 (26.4%)	55 (24.3%)	10 (50%)	0.01
NYHA class 1–2	148 (60.2%)	135 (59.7%)	13 (65%)	0.64
NYHA class 3–4	35(14.2%)	30 (13.3%)	5 (25.0%)	0.15
AF characteristics				
Paroxysmal	96 (39.0%)	94 (41.6%)	2 (10%)	0.006
Persistent	71 (28.9%)	66 (29.2%)	5 (25%)	0.7
Permanent	79 (32.1%)	66 (29.2%)	13 (65%)	0.001
CHA_2_DS_2_-VASc	4 (3–5)	4 (3–5)	5 (4–5)	0.04
HAS-BLED	1 (1–1)	1 (1–1)	1 (1–1)	0.26
Cardiovascular risk factors and comorbidities				
HTN	215 (87.4%)	198 (87.6%)	17 (85%)	0.74
IHD	66 (26.8%)	64 (28.3%)	2 (10%)	0.08
Prior MI	15 (6.1%)	14 (6.2%)	1 (5%)	0.83
TIA/Stroke	34 (13.8%)	32 (14.2%)	2 (10%)	0.60
Diabetes mellitus	63 (25.6%)	58 (25.7%)	5 (25%)	0.94
Dyslipidemia	197 (80.1%)	186 (82.3%)	11 (55%)	0.003
Obesity	90 (36.6%)	86 (38.0%)	4 (20%)	0.11
Dementia	9 (3.7%)	9 (4%)	0 (0%)	0.36
Infection	31 (12.8%)	28 (12.5%)	3 (15.8%)	0.68
HR, bpm	84.3 ± 23.1	84.2 ± 23.3	84.4 ± 20.7	0.75
Echocardiographic characteristics				
LAD, mm	43.5 ± 5.5	43.3 ± 5.5	46.1 ± 5.4	0.03
LVEDD, mm	48.1 ± 5.9	48.3 ± 5.8	45.5 ± 6	0.06
LVESD, mm	31.3 ± 5.4	31.5 ± 5.4	29.0 ± 5.4	0.06
RAD, mm	40.5 ± 6.4	39.8 ± 5.6	48.8 ± 10.2	<0.001
RVD, mm	32.7 ± 5.7	32.1 ± 5.0	39.1 ± 8.6	<0.001
sPAP, mmHg	28.8 ± 6.9	28.2 ± 6.8	35.7 ± 3.6	<0.001
LVEF, %	55.9 ± 4.2	56.0 ± 4.3	54.7 ± 4.3	0.19
Laboratory characteristics				
NT-proBNP, pg/mL	1086 (539–1837)	957 (486–1784)	1686 (1300–3763)	0.003
eGFR, mL/min/1	75.7 (57.6–92.3)	77.1 (59.9–93.0)	55.34 (51.7–75.2)	0.007
HB, g/dL	13.6 (12.4–14.6)	13.6 (12.5–14.7)	12.6 (11.3–14)	0.30
ADHF, acute decompensated heart failure; AF, atrial fibrillation; eGFR, estimated glomerular filtration rate; LVEF, left ventricle ejection fraction; HB, hemoglobin; HF, Heart failure; HR, heart rate; HTN, arterial hypertension; IHD, ischemic heart disease; LAD, left atrium diameter; LVEDD, left ventricular end-diastolic diameter; LVESD, left ventricular end-systolic diameter; MI, myocardial infarction; NYHA, New York Heart Association; RAD, right atrium diameter; RVD, right ventricular diameter; TIA, transient ischemic attack; TR, tricuspid regurgitation; sPAP, systolic pulmonary arterial pressure.* Patients with non-severe AF-TR; ** Patients with severe AF-TR				

**Table 2:** Echocardiographic characteristics stratified by type of AF.

	Paroxysmal AF N = 96	Persistent AF N = 71	Permanent AF N = 79	*p* for Trend
Severe TR, n	2	5	13	0.0006
RAD, mm	37.4 ± 4.5	41.2 ± 5.7	43.8 ± 7.3	<0.001
RVD, mm	30.9 ± 4.7	33.3 ± 4.9	34.2 ± 6.8	0.0004
sPAP, mmHg	26.8 ± 7.1	28.9 ± 6.6	31.3 ± 6.3	<0.001
LVEF, %	56.5 ± 4.4	56.0. ± 3.9	55.2 ± 4.5	0.13
LAD, mm	41.5 ± 5.6	43.7 ± 4.7	45.9 ± 5.2	<0.001
LVEDD, mm	48.6 ± 6.4	47.2 ± 5.7	48.2 ± 5.2	0.32
LVESD, mm	31.9 ± 5.4	30.7 ± 5.6	31.1 ± 5.2	0.36

AF, atrial fibrillation; LAD, left atrium diameter; LVEDD, left ventricular end-diastolic diameter; LVESD, left ventricular end-systolic diameter; n, number; RAD, right atrium diameter; RVD, right ventricular diameter; Spap, systolic pulmonary arterial pressure; TR, tricuspid regurgitation.

**Table 3:** Predictors of severe AF-TR—univariate analysis.

	OR (95% CI)	*p* Value
ADHF at admission	3.10 (1.2–7.8)	0.01
Permanent AF	6.40 (1.5–28.3)	0.005
	**AUC (95% CI)**	***p* Value**
Age	0.70 (0.58–0.81)	0.003
LAD	0.67 (0.50–0.80)	0.015
RAD	0.81 (0.71–0.91)	<0.001
RVD	0.80 (0.70–0.90)	<0.001
sPAP	0.82 (0.75–0.90)	<0.001
NT-proBNP	0.76 (0.67–0.85)	0.045
eGFR	0.68 (0.57–0.80)	0.007
CHA_2_DS_2_-VASc score	0.63 (0.52–0.74)	0.05

ADHF, acute decompensated heart failure; AF, atrial fibrillation; AUC, area under ROC curve; eGFR, estimated glomerular filtration rate; LAD, left atrium; OR, odds ratio; RAD, right atrium diameter; RVD, right ventricular diameter; sPAP, systolic pulmonary arterial pressure.

**Table 4:** Independent predictors of severe AF-TR.

	HR (95% CI)	*p* Value
RAD	1.11 (1.03–1.20)	0.005
sPAP	1.20 (1.03–1.38)	0.015
NTproBNP	2.37 (1.10–5.10)	0.026

HR, hazard ratio; RAD, right atrium diameter; sPAP, systolic pulmonary arterial pressure. Parameters included in the multivariable logistical regression without independent predictive value: acute decompensated heart failure, permanent atrial fibrillation, left atrium diameter, right ventricular diameter, estimated glomerular filtration rate, CHA_2_DS_2_-VASc score.

**Table 5:** Univariate analysis—predictors of mortality in AF patients.

	OR (95% CI)	*p* Value
Severe AF-TR	4.37 (1.41–13.57)	0.005
ADHF	4.32 (1.72–10.83)	<0.001
NYHA class III/IV	5.74 (2.21–14.93)	<0.001
TIA/Stroke	2.81 (1.11–7.85)	0.04
Dementia	6.08 (1.40–26.34)	0.006
Infection	5.27 (2.02–14.21)	<0.001
	**AUC (95% CI)**	***p*** **Value**
Age	0.77 (0.66–0.88)	<0.001
RVD	0.64 (0.50–0.78)	0.05
LVEF%	0.69 (0.56–0.81)	0.009
NT-proBNP	0.80 (0.71–0.90)	<0.001
eGFR	0.72 (0.61–0.84)	0.01
HB	0.63 (0.50–0.76)	0.05

ADHF, acute decompensated heart failure; AUC, area under the ROC curve; eGFR, estimated glomerular filtration rate; HB, hemoglobin; LVEF, left ventricular ejection fraction; NYHA, New York Heart Association; RR, risk ratio; RVD, right ventricular diameter; TIA, transient ischemic attack; TR, tricuspid regurgitation; sPAP, systolic pulmonary arterial pressure.

**Table 6:** Cox proportional hazards analysis—predictors of mortality in AF patients.

	HR (95% CI)	*p* Value
Severe AF-TR	5.4 (1.1326.17)	0.035
Dyspnea at mild effort or at rest	3.90 (1.29–11.82)	0.016
Infection	3.79 (1.27–11.26)	0.017
Age	1.07 (1.01–1.14)	0.029
LVEF	0.82 (0.72–0.95)	0.008

AF-TR, atrial functional tricuspid regurgitation; HR, hazard ratio; LVEF, left ventricular ejection fraction. Parameters included in the multivariable Cox proportional hazards analysis without independent predictive value: acute decompensated heart failure, transient ischemic attack or stroke, dementia, right ventricular diameter, NT-proBNP, estimated glomerular filtration rate, hemoglobin levels.

## Data Availability

Data used in this study may be provided by the authors upon reasonable request.
